# Transition from Cyclosporine-Induced Renal Dysfunction to Nephrotoxicity in an *in Vivo* Rat Model

**DOI:** 10.3390/ijms15058979

**Published:** 2014-05-20

**Authors:** José Sereno, Paulo Rodrigues-Santos, Helena Vala, Petronila Rocha-Pereira, Rui Alves, João Fernandes, Alice Santos-Silva, Eugénia Carvalho, Frederico Teixeira, Flávio Reis

**Affiliations:** 1 Laboratory of Pharmacology & Experimental Therapeutics, Institute for Biomedical Imaging and Life Sciences (IBILI), Faculty of Medicine, University of Coimbra, Coimbra 3000-548, Portugal; E-Mails: jose6sereno@hotmail.com (J.S.); jfernandes@email.com (J.F.); fredjt@ci.uc.pt (F.T.); 2 Institute for Nuclear Sciences Applied to Health (ICNAS), University of Coimbra, Coimbra 3000-548, Portugal; 3 Institute of Immunology, Faculty of Medicine, University of Coimbra, Coimbra 3004-504, Portugal; E-Mail: psantos@ci.uc.pt; 4 Immunology and Oncology Laboratory, Center for Neurosciences and Cell Biology, Coimbra 3004-504, Portugal; 5 Agrarian School of Viseu (ESAV), Polytechnic Institute of Viseu, Viseu 3500-606, Portugal; E-Mail: hvala2@gmail.com; 6 Educational, Technologies and Health Study Center, Polytechnic Institute of Viseu, Viseu 3500-606, Portugal; 7 Research Center for Health Sciences, Beira Interior University, Covilhã 6201-506, Portugal; E-Mail: petronila@live.com.pt; 8 University Nephrology Unit, Faculty of Medicine, University of Coimbra, Coimbra 3004-504, Portugal; E-Mail: ruimbalves@hotmail.com; 9 Biochemistry Department, Pharmacy Faculty, Porto University, Porto 4050-313, Portugal; E-Mail: assilva@ff.up.pt; 10 Institute for Molecular and Cellular Biology, Porto University, Porto 4150-180, Portugal; 11 Insulin Resistance & Adipocyte Group, Center for Neuroscience and Cell Biology, Coimbra 3004-504, Portugal; E-Mail: genita67@gmail.com; 12 The Portuguese Diabetes Association (APDP), Lisbon 1250-203, Portugal

**Keywords:** Cyclosporin A, transition from dysfunction to nephrotoxicity, biomarkers, fibrosis, proliferation, animal model

## Abstract

Cyclosporin A (CsA), a calcineurin inhibitor, remain the cornerstone of immunosuppressive regimens, regardless of nephrotoxicity, which depends on the duration of drug exposure. The mechanisms and biomarkers underlying the transition from CsA-induced renal dysfunction to nephrotoxicity deserve better elucidation, and would help clinical decisions. This study aimed to clarify these issues, using a rat model of short- and long-term CsA (5 mg/kg bw/day) treatments (3 and 9 weeks, respectively). Renal function was assessed on serum and urine; kidney tissue was used for histopathological characterization and gene and/or protein expression of markers of proliferation, fibrosis and inflammation. In the short-term, creatinine and blood urea nitrogen (BUN) levels increased and clearances decreased, accompanied by glomerular filtration rate (GFR) reduction, but without kidney lesions; at that stage, CsA exposure induced proliferating cell nuclear antigen (*PCNA*), transforming growth factor beta 1 (*TGF-β1*), factor nuclear kappa B (*NF-κβ*) and Tumor Protein P53 (*TP53*) kidney mRNA up-regulation. In the long-term treatment, renal dysfunction data was accompanied by glomerular and tubulointerstitial lesions, with remarkable kidney mRNA up-regulation of the mammalian target of rapamycin (*mTOR*) and the antigen identified by monoclonal antibody Ki-67 (*Mki67*), accompanied by mTOR protein overexpression. Transition from CsA-induced renal dysfunction to nephrotoxicity is accompanied by modification of molecular mechanisms and biomarkers, being mTOR one of the key players for kidney lesion evolution, thus suggesting, by mean of molecular evidences, that early CsA replacement by mTOR inhibitors is indeed the better therapeutic choice to prevent chronic allograft nephropathy.

## 1. Introduction

Calcineurin inhibitors (CNIs), such as Cyclosporine A (CsA), remain pivotal immunosuppressive drugs to prevent allograft rejection and its introduction in clinical practice led to a significant improvement in post-transplant survival [1,2]. CsA binds to cyclophilin with the synergistic action of calcium and suppresses activation of the calcium-dependent phosphatase calcineurin, thus inhibiting interleukin-2 release and blocking T cell activation [3]. However, the clinical use of CsA is often limited by severe side-effects, including hypertension and nephrotoxicity [4–6]. Renal dysfunction is an independent risk factor for graft loss and mortality after kidney transplantation (KTx) and cardiovascular/cardiorenal disease is the main cause of dead post-KTx [7–9]; thus, extended long-term graft survival has not been completely achieved.

The recognition of these serious adverse effects sparked interest in CsA-sparing strategies [10]: CsA avoidance is associated with high acute rejection rates and is not an option; dose reduction is associated with a modest improvement in renal function, but CsA-induced nephropathy is progressive over time when exposure is maintained; minimization protocols are the current preferred therapy, including the conversion from CsA to other drugs, specially to Sirolimus (SRL), an inhibitor of the mammalian target of rapamycin (mTOR) [11,12]. Late conversion from CsA to SRL has achieved variable results, possibly because withdrawal was attempted after the kidney damage was already too extensive. Early conversion, on the other hand, prior to significant graft damage, has generally improved creatinine clearance and markers of fibrosis, and decreased chronic allograft lesions, but the issue is far from being consensual, as the Convert trial has demonstrated [13–16]. The major question nowadays concerning the protocols of immunotherapy is to find the most adequate duration for CsA exposure and the proper moment for replacement by other less nephrotoxic drugs, without compromising the graft by a rejection episode. The answers to these questions largely rely on the characterization of precise mechanism and feasible biomarkers underlying transition from renal dysfunction to nephrotoxicity and experimental studies are crucial to improve the knowledge of this translational issue of clinical relevance.

Early diagnosis of nephropathy can greatly improve patient prognosis, but the initial stages of CsA-induced nephropathy are largely asymptomatic, making early diagnosis difficult [17]. Since the current diagnostic techniques employed to detect CsA-evoked nephropathy seem to be unsatisfactory, the identification of novel, early disease indicators is currently a major research focus. CsA-induced nephropathy seems to be linked with changes on mechanisms related with oxidative stress, apoptosis and proliferation/fibrosis [18–23]; putatively modulated by influence on renal tissue gene expression [23–25]. Identifying drug safety liabilities or predictive biomarkers for drug-induced organ damage is of great value. Drug safety evaluation has mainly been based on biochemical and histopathological data, but transcriptional profiling has the promise of being able to accurately and earlier detect toxicity. In fact, gene expression changes may improve our understanding on the mechanisms of drug-induced toxicity [26,27].

In a preliminary study [28] we found that rats treated with CsA for 6 weeks present changes on renal gene expression profile of inflammatory and proliferative mediators, in a stage when glomerular and tubule-interstitial lesions were yet absent and only some vascular lesions were present, although in a slight stage. These findings have strongly recommended the elucidation of those mechanisms in two particular important phases of disease evolutions: (a) when the impaired kidney (renal dysfunction) remains histologically unchanged and (b) after the development of structural changes (lesions). Thus, our present study was performed in order to clarify the pathways and putative biomarkers involved in transition from CsA-induced renal dysfunction to nephrotoxicity in an animal model. To do so, short- and long-term CsA treatments (3 and 9 weeks, respectively) were now compared using serum, urine and renal tissue samples and analyzing biochemical, histological and gene expression markers, which are possible/probable candidates to act as players in the evolution of CsA-evoked nephropathy.

## 2. Results

### 2.1. Blood, Serum, Urine and Kidney Tissue Biochemical Data

In this study, a rat CsA dose that mimics de trough blood concentration of CsA found in humans under CsA immunosuppressive therapy was practized. Therefore, the trough blood concentration of CsA obtained using the dose of 5 mg/kg/day in the rat (367.0 ± 45.5 ng/mL) was within the range achieved in humans.

**Figure 1.**
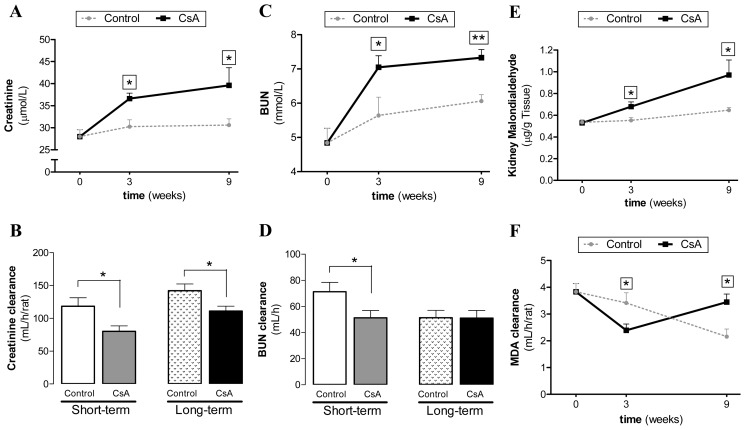
Serum, urine and kidney markers of renal function. Creatinine serum levels (**A**) and clearance (**B**); blood urea nitrogen (BUN) levels (**C**) and clearance (**D**); kidney lipid peroxidation evaluated by the malondyaldehyde content (**E**); malondyaldehyde clearance (**F**) throughout the short- and long-term Cyclosporin A (CsA) treatments. Values are mean ± SEM. * *p* < 0.05 and ** *p* < 0.01 *vs.* the Control group.

Serum creatinine and blood urea nitrogen (BUN) levels significantly increased (*p* < 0.05) in the short-term (3 weeks) CsA-treated rats, accompanied by a significantly decreased (*p* < 0.05) creatinine and BUN clearance (Figure 1). Long-term CsA treatment further aggravated the serum creatinine and BUN levels, accompanied by a creatinine clearance decrease (*p* < 0.05). The glomerular filtration rate (GFR) decreased after both 3 and 9 weeks of treatment (*p* < 0.05, data not shown). In addition, CsA-evoked kidney lipid peroxidation (malondialdheyde (MDA) levels) increased (*p* < 0.05) in the short-term treatment, accompanied by reduction of MDA clearance (*p* < 0.05). After 9 weeks of CsA exposure, both kidney MDA levels and MDA clearance significantly increased (Figure 1). Although both CsA-treated groups (3 and 9 weeks) showed statistically significant differences when compared with the corresponding controls, the AUC values do not differ (*p* = 0.6111) between the two treatments, which might be due to the reversal of MDA clearance profile between 3 and 9 weeks (Figure 1F).

### 2.2. Kidney Histological Data

**Figure 2.**
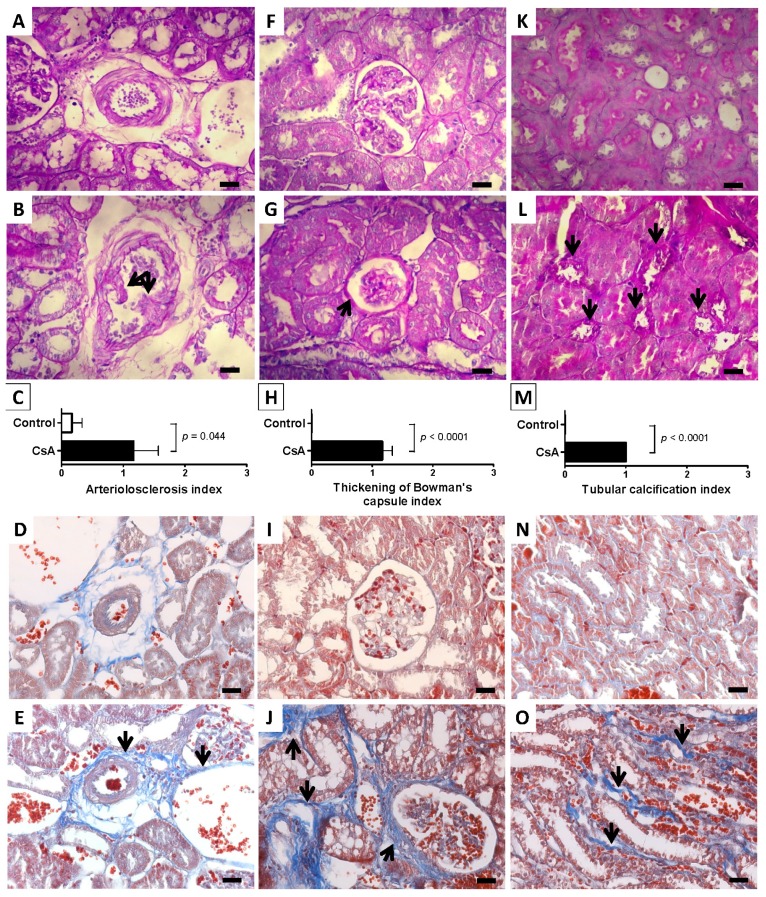
Kidney lesions. Representative photomicrographs of kidney histological sections stained with periodic acid of Schiff (PAS), for Control (**A**,**F**,**K**) and CsA (**B**,**G**,**L**) groups in the long-term CsA treatment model; (**A**) represents a normal kidney arteriole from the control group and (**B**) an arteriolosclerosis lesion present in all the rats treated with CsA, indicated by the two arrows; (**F**,**G**) represent a normal capsule and a thickening Bowman’s capsule (black arrow) from the CsA group, respectively; (**K**,**L**) show a normal tubules and tubular calcification (black arrows) in control and CsA-treated rats, respectively; (**C**,**H**,**M**) represent the index of each kidney lesion for the Control and CsA groups; Representative photomicrographs of kidney histomorphologic sections with Masson’s trichrome staining for Control (**D**,**I**,**N**) and CsA (**E**,**J**,**O**) groups in the long-term CsA treatment model, showing the pattern of collagen deposition (blue color); (**D**,**I**,**N**) show normal vascular, glomerular and tubulointerstitial regions of the control rats; (**E**) presents an arteriolosclerosis lesion and collagen deposition around vessels (black arrows) CsA-treated rats; (**J**,**O**) show collagen deposition around Bowman’s capsule and tubules (black arrows) and tubule-interstitial fibrosis (black arrows) from the CsA-treated group, respectively. Each bar represents 25 μm.

Nephrotoxicity was confirmed by two independent pathologists, which have characterize the lesions by scoring each vascular, glomerular and tubular lesion, using kidney slices stained with haematoxylin and eosin (H&E) and periodic acid of Schiff (PAS). Despite the significantly increased markers of renal function, the short-term CsA treatment was unable to promote significant histological changes on the kidney tissue when compared with the control. However, 9 weeks of CsA exposure promoted important changes on the kidney (vessels, glomeruli and tubules) structure, suggesting nephrotoxicity development. The main changes encountered compared with the normal controls are represented on Figure 2.

In long-term CsA exposure, hyperemia, arteriolar vacuolization and vascular congestion were identified (statistically increased *vs.* the Control) in the rat kidneys (data not shown), but arteriolosclerosis was the most important vascular lesion observed (Figure 2B) when compared with the control animals (Figure 2A). Regarding glomerular lesions, the major findings (*p* < 0.05) were mesangial expansion, hyalinosis of vascular pole and thickening of Bowman’s capsule (Figure 2G), when compared with the control rat kidneys (Figure 2F). In addition to tubular vacuolization, other tubular lesions were encountered, including hyaline cylinders, inflammatory infiltrate and tubular calcification (Figure 2L) *vs.* the normal profile found in the control rats (Figure 2K). Figure 2C,H,M show the statistically significant differences encountered in terms of arteriolosclerosis, thickening of Bowman’s capsule and tubular calcification in the kidneys of long-term CsA-treated animals, when compared with the control ones. Renal fibrosis was revealed using Masson’s Trichrome staining. In the kidneys from vehicle-treated rats, collagen deposition (blue color) was rare in the glomeruli, and a small amount of staining appeared in the outer borders tubules and around the vessels (Figure 2D). After nine weeks of CsA treatment, strong blue staining in the outer borders of tubular cells (cortex and medulla) was well identified, representing widespread interstitial fibrosis (Figure 2E,J). Thickening of Bowman’s capsule also occurred in some glomeruli (Figure 2E). Around the vessels, CsA was able to induce even higher collagen deposition (Figure 2O).

### 2.3. Kidney Gene Expression Data

**Figure 3.**
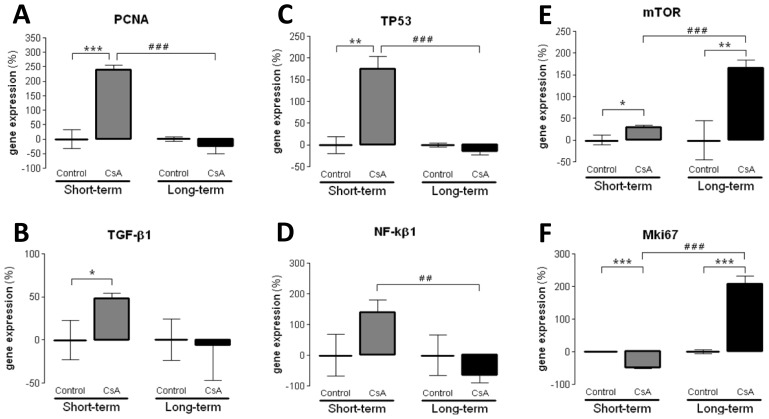
Kidney gene (mRNA) expression of markers of proliferation. *PCNA* (**A**); *TGF-β1* (**B**); *TP53* (**C**); *NF-κB* (**D**); *mTOR* (**E**) and *Mki67* (**F**) for the control and CsA treatments, in the short- and long-term models. Values are mean of % of the control ± SEM. * *p* < 0.05, ** *p* < 0.01 and *** *p* < 0.001 *vs.* the Control group of each model; ^##^ *p* < 0.01 and ^###^ *p* < 0.001 *vs.* the CsA-acute model. *Mki67*, antigen identified by monoclonal antibody Ki-67; *mTOR*, mammalian target of rapamycin; *NF-κB1*, nuclear factor kappa B; *PCNA*, proliferating cell nuclear antigen; *TGF-β1*, transforming growth factor beta 1; *TP53*, tumor protein p53.

**Figure 4.**
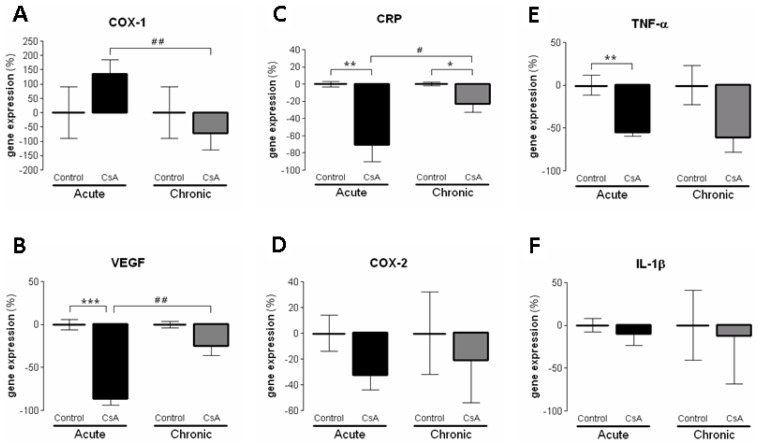
Kidney gene (mRNA) expression of markers of inflammation and angiogenesis. *COX-1* (**A**); *VEGF* (**B**); *CRP* (**C**); *COX-2* (**D**); *TNF-α* (**E**) and *IL-1β* (**F**) for the control and CsA treatments, in the short- and long-term models. Values are mean of % of the control ± SEM. * *p* < 0.05, ** *p* < 0.01 and *** *p* < 0.001 *vs.* the Control group of each model; ^#^ *p* < 0.05 and ^##^ *p* < 0.01 *vs.* the CsA-acute model. *COX-1*, ciclooxigenase-1; *COX-2*, ciclooxigenase-2; *CRP*, C-reactive protein; *IL-1β*, interleukin 1 beta; *TNF-α*, tumor necrosis factor alpha.

Several markers of proliferation, fibrosis, inflammation and angiogenesis were evaluated in terms of kidney mRNA expression in both the short- and long-term CsA-treated rats in comparison to controls. After 3 weeks of CsA treatment, a significant down-regulation of the antigen identified by the monoclonal antibody Ki67 (*MKi67*) was found (Figure 3). Furthermore, there was a significant over-expression of proliferating cell nuclear antigen (*PCNA*) and tumor protein p53 (*TP53*), accompanied by a slight increase in the expression of transforming growth factor beta 1 (*TGF-β1*) and nuclear factor kappa B (*NF-κB*). On the other hand, in the long-term CsA treatment, *PCNA*, *TP53*, *TGF-β1* and *NF-κB* expression were unchanged *vs.* the control. However, and surprisingly, both *MKi67* and *mTOR* were remarkably over-expressed in contrast to what was observed with the short-term CsA exposure (Figure 3). Concerning markers of inflammation and angiogenesis, both the short- and long-term CsA-treated rats showed a down-regulation of cyclooxygenase-1 and cyclooxygenase-2 (*COX-1* and *COX-2*), *C*-reactive protein (*CRP*), Tumor necrosis factor alpha (*TNF-α*) and vascular endothelial growth factor (*VEGF*) (Figure 4).

### 2.4. Kidney Mammalian Target of Rapamycin (mTOR) Protein Expression

**Figure 5.**
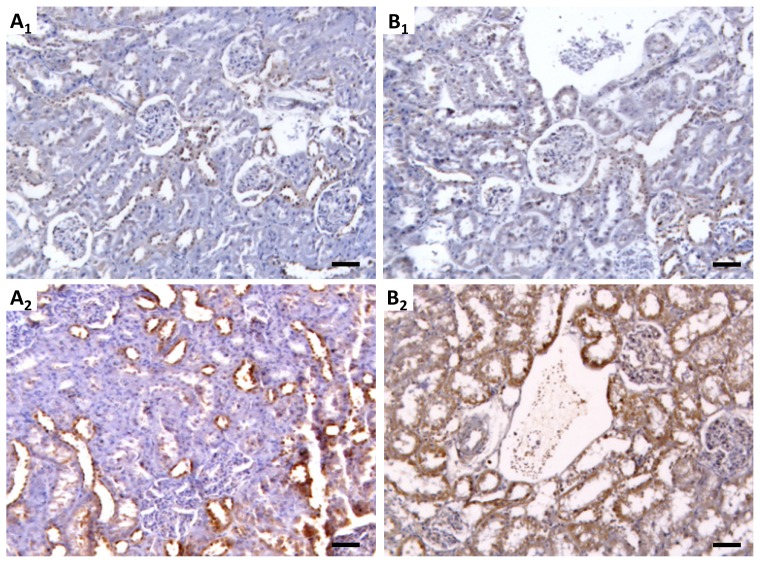

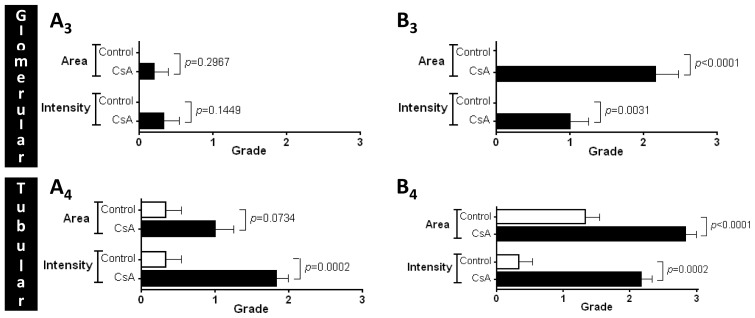
Kidney mTOR protein expression. Immunohistochemistry pictures from control (**A_1_**,**B_1_**) and CsA-treated (**A_2_**,**B_2_**) rats, after short- and long-term treatments (3 and 9 weeks, respectively), and staining area and intensity in the glomerular (**A_3_**,**B_3_**) and tubule-interstitial region (**A_4_**,**B_4_**). Each bar represents 50 μm.

In agreement with the mRNA results, mTOR protein expression was increased in the CsA-treated rats, with a moderate increment after the short-term treatment, with grade 1 staining area (25%–50%) and grade 2 intensity (Figure 5A_4_) in the tubular region, without significant area and staining intensity in the glomerular field (Figure 5A_3_). More pronounced expression under a long-term CsA treatment was observed, with >75% of staining area (grade 3) and grade 1 intensity (Figure 5B_3_) in the glomeruli and with >75% of staining are (grade 3) and grade 2 intensity (Figure 5B_4_) in the tubulointerstitial region, both compared with the corresponding control rats of the 3 and 9 weeks, both demonstrating no staining (Figure 5A_1_,B_2_).

## 3. Discussion

Despite the substitute therapeutics that has been sought, calcineurin inhibitors remain the most effective and widely used immunosuppressive agents post-transplantation, especially in the initial period [29]. The management of CsA-induced nephropathy and other CsA-evoked side-effects includes therapeutic strategies devoted to minimize its use of replace by other putatively less nephrotoxic drugs, such as Sirolimus, an mTOR inhibitor, as well as to control dyslipidemia and hypertension, usually present. Until the precise molecular mechanisms underlying CsA-induced nephrotoxicity are fully clarified, the choice of the most appropriate moment to promote the substitution (early or late conversion) and the choice of the best anti-hypertensive/renoprotective drugs, typically needed, will remain empirical options that consider the following aspects: the control of blood pressure, the side-effects of drugs per se and the potential interferences with immunosuppressive therapy. Under these conditions, the renal and cardiovascular impairment will progress, thus originating more serious consequences: graft loss and/or dead due to cardiorenal complications. In the last decades, there has been a clear change in post-transplantation management, with particular attention to the long-term kidney function and overall quality of life improvement. In this context, the search for the precise molecular mechanism underlying short- *vs.* long-term CsA-induced nephropathy is essential and depends largely on experimental studies.

Although CsA-induced nephrotoxicity has been studied for several years, the underlying mechanisms remain to be elucidated and the problem unsolved [30]. CsA immunosuppressive mechanism of action rely on inhibition of Nuclear factor of activated T-cells (NFAT), a CsA-sensitive transcription factor implicated in cytokine gene induction, which is essential for interleukin 2 (IL-2) formation and further maturation of T-lymphocytes required for immune response [31]. However, the NFAT isoforms are not T cell specific, and inhibition of this pathway by CsA gives rise to toxicity beyond immunosuppression [32]. Calcineurin, a Ca^2+^/calmodulin-dependent protein phosphatase, is an important signaling molecule and has been implicated in hypertrophy of a variety of cell types [33]. However, not much is yet known concerning the influence of short- *vs.* long-term CsA exposure on kidney gene expression, particularly of several important mediators of fibrosis/proliferation, which are key pathways in the deterioration of renal tissue, as viewed in different animal models of kidney disease [34–36]. Since the duration of CsA-treatment accompanies its toxicity, we intended to contribute for the elucidation of the influence of a short- *vs.* long-term CsA treatment on kidney function, structure and gene expression.

Regarding biochemical characterization, we found that serum markers of renal dysfunction (creatinine and BUN concentrations) were already significantly increased after 3 weeks of CsA treatment, further increasing in the long-lasting CsA exposure. Similar variation was found for kidney lipid peroxidation and lesions. Therefore, despite the increased contents of creatinine and BUN (accompanied by decreased creatinine, BUN and malondialdehyde (MDA) clearances), as well as the GFR, after the third week of CsA therapy, slight or even absent changes on tissue structures were found. However, after the long-term CsA exposure, significant glomerular, tubular and vascular lesions were observed. In our study, the renal markers used in clinical practice (GFR, creatinine and BUN contents and clearances) were impaired in the CsA-treated rats. Additionally, we observed an interesting variation of MDA levels between 3 and 9 weeks of CsA treatment. The significant increase in the MDA clearance observed in our model raises some questions. In 1989, Knight and colleagues detected high MDA levels in urine of transplanted patients [37], but they were unable to find the cause/effect of these observations, suggesting that it could be explained by: (a) renal lipid peroxidation directly related to the cyclosporine/azathioprine therapy; (b) drug-induced or other nephrotoxicity by an alternative mechanism with secondary lipid peroxidation; (c) increased lipid peroxidation owing to an immunologic response to the kidney graft; or (d) a combination of these possibilities. Our data suggests that MDA clearance could be a predictive marker of CsA-nephrotoxicity, since the increased MDA clearance appears at the same time-point as the first kidney lesions. Oxidative stress can promote the formation of a variety of vasoactive mediators [38] that can affect renal function directly by causing renal vasoconstriction or decreasing the glomerular capillary ultrafiltration coefficient, thus reducing the glomerular filtration rate. The current diagnostic techniques employed to detect CsA-evoked nephrotoxicity are still inadequate. The estimated GFR can be an insensitive indicator, since it depends on various factors, including blood factors and age. Creatinine and BUN clearances have poor diagnostic value [39]. Moreover, the relationship between proteinuria and CsA-induced nephrotoxicity is complex, limiting its use as an early biomarker [10]. Lipid peroxidation occurs as a result of multi-unsaturated lipids reacting with oxidizing agents, promoting oxidative stress in the kidney structures. Urinary MDA reflects the presence of renal damage, which may be the cause or the consequence of lipid peroxidation. Therefore, the correlation between MDA clearance and kidney lesion grade could be a good strategy to identify early CsA-evoked nephrotoxicity. Overall, we suggest that the reversal of MDA clearance profile between 3 and 9 weeks might be further viewed as a biomarker of CsA damage, in the sense that is coincident with the transition from CsA dysfunction to nephrotoxicity. However, to strength this possibility a longer treatment is needed in order to confirm if this MDA clearance rise continues overtime. Additionally, further research should clarify if aging, even in control animals, is associated with a reduced MDA clearance and accumulation in the kidney tissue, as our data suggests.

Concerning the molecular mechanisms underlying short- and long-term renal dysfunction/nephrotoxicity, previous reports have suggested a close link between severe glomerular and tubular lesions (including atrophy, fibrosis, inflammation and sclerosis) and increased mediators of inflammatory response, with activation of NF-κB and increased TGF-β1 release, which then causes nephropathy mediated by fibrosis and apoptosis of renal cells [40–42]. However, old and new versions of the putative mechanisms have been suggested, but the main question remains unsolved. In our animal model, the short-term CsA treatment was mainly associated with up-regulation of mediators of fibrosis and proliferation, with over-expression of *TGF-β1* and *PCNA*, accompanied by a slight increase in the expression of *NF-κB*, which has been identified as a key mediator of inflammation and fibrosis [41,42]. However, these changes were accompanied by putative compensatory response, since markers of inflammation (including *COX2*, *TNF-α* and *CRP*), as well as, cellular proliferation (*MKi67*) and angiogenesis (*VEGF*) were downregulated, perhaps responsible for the attenuation of the cytotoxic effects in the short-term CsA exposure. The over-expression of *NF-κB* and *TP53* might be included in that compensatory response, since they can inhibit mTOR [43]. Nevertheless, during prolonged CsA exposure nephrotoxicity evolves, as viewed by the degree of histological lesions, which seems to be associated with other molecular pathways and mediators. In fact, there was a significant over-expression of *MKi67*, in opposition to what was found after the short-term treatment, suggesting a depletion of counter-regulatory responses; this effect was accompanied by a parallel increase in gene and protein *mTOR* expression, a serine/threonine protein kinase with a key role in regulating cell growth, proliferation, motility, survival, protein synthesis and transcription [44]. Our data suggests that long-term CsA treatment implicates proliferation pathways and mediators evolving more sharply than others, such as the Mki67 that enters in all phases of cell cycle and the mTOR that is essential to cell survive and proliferation expression.

Complete restoration of renal morphology and function can occur after acute kidney injury induced by ischemic or toxic injury. Renal regeneration depends, in part, on the ability of the remaining viable tubular cells to proliferate and restore the injured tubular epithelium [41]. As Lieberthal *et al.* demonstrated, mTOR plays an important role in mediating the process of regeneration and recovery, depending on the kidney damage extension [45]. Moreover, mTOR activity is low or absent in the normal kidney but increases markedly after acute kidney injury. In agreement, mTOR inhibition has been associated with amelioration of kidney fibrosis, glomerulosclerosis and interstitial inflammation, having an important role in renal disease [45–47]. Concerning transplantation, mTOR inhibitors have been used to replace CsA, because of its reduced putative side-effects, including nephrotoxicity [12,13]. Our results reinforce the rationale for the early substitution of CsA by mTOR inhibitors, not only because longer CsA exposure is notoriously more deleterious, promoting structural kidney deterioration, but also because mTOR over-expression seems to be a feature of the chronic CsA exposure. Recently, Luo *et al.* [22] showed that rapamycin is less fibrogenic than CsA; thus, early detection of CsA-induced nephropathy and proper substitution for more adequate drugs, which seem to be the mTOR inhibitors, will greatly reduce the risk of chronic allograft nephropathy and improve the outcomes in transplanted patients. In chronic kidney disease, rapamycin also slows the progression of renal fibrosis and delays the onset of renal failure, through reduction of glomerular hypertrophy, decrease of proinflammatory and profibrotic cytokines production and decline of interstitial inflammation [45].

This study presents new findings concerning the mechanisms underlying transition from CsA-induced renal dysfunction to nephrotoxicity which remains a clinically relevant issue in immunotherapy after organ transplantation, as well as, in patients under immunosuppressive treatment for autoimmune disorders. The absence of alloimmune response in this model of native kidneys is recognized as a limitation with possible interference on the outcome, deserving further confirmation. However, the results are also interesting and show applicability in other clinical cases of CsA-induced nephrotoxicity without allograft transplantation, such as psoriasis, rheumatoid arthritis, systemic lupus erythematosus and inflammatory bowel disease, among other indications. Future work will assess the correlation between the gene and the protein expression profiles, in order to assess if there is a relationship between the genetic pathways and the protein production in the dysfunctional and in the damaged kidney, as well as, to confirm the nature of the biphasic response underlying transition from CsA-induced dysfunction to nephropathy.

## 4. Experimental Section

### 4.1. Animals and Treatments

Twenty four male *Wistar* rats (Charles River Lab. Inc., Barcelona, Spain), eleven weeks of age, were maintained as above mentioned, subjected to 12 h dark/light cycles and given standard laboratory rat chow (IPM-R20, Letica, Barcelona, Spain) and free access to tap water. The animals were divided into two groups: short- and long-term CsA (5 mg/kg/day of Sandimun Neoral^®^) exposure, of 3 and 9 weeks respectively *vs.* the corresponding control groups (vehicle), with an *n* = 6 per group. Treatments were performed by oral gavage. Body weight was monitored throughout the treatment period. Cyclosporine (Sandimun Neoral^®^) was obtained from Novartis Farma Produtos Farmacêuticos SA (Sintra, Portugal). Animal experiments were conducted according to the European Council Directives on Animal Care and to the National Authorities, and the study received approval by the local ethics committee (Organ Responsible for Animal Welfare) for research animal studies. Animals were maintained in certificated rat vivarium, in adequate individual ventilated cages, with adequate measures of welfare and reduction of suffering during procedures, including environment enrichment and anesthesia/analgesia.

### 4.2. Sample Collection and Preparation

Before the treatments (T0) and at the end of the acute and chronic protocols, the rats were injected with intraperitoneal anesthesia with 2 mg/kg bw of a 2:1 (*v*/*v*) 50 mg/mL Ketamine (Ketalar^®^, Parke-Davis, Pfizer Laboratories Lda, Seixal, Portugal) solution in 2.5% chlorpromazine (Largatil^®^, Rhône-Poulenc Rorer, Vitória laboratories, Amadora, Portugal). Blood samples were immediately collected by venipuncture from the jugular vein in needles with no anticoagulant (for serum samples collection) or with appropriated anticoagulant (ethylenediamine tetraacid (EDTA)) for blood cell analysis. At the final time, the rats were sacrificed by cervical dislocation, and the kidneys were immediately removed, weighted and stored in RNA-stabilizer reagent for gene expression determinations, frozen in nitrogen for lipid peroxidation assays and pre-fixed with formaldehyde for histopathological analysis.

### 4.3. Blood, Serum and Urine Data

Blood: CsA blood concentrations were assessed by immunoassay using automatic methods (Flex reagent) and equipment (Dimension^®^RxL, Siemens, Erlangen, Germany).

Serum: Creatinine and blood urea nitrogen (BUN) concentrations were evaluated as renal function indexes, and assessed using automatic validated methods and equipment (Hitachi 717 analyzer, Roche Diagnostics Inc., Boston, MA, USA).

Urine: The animals were housed in metabolic cages during 24 h and received tap water and food *ad libitum*. The urine concentration of creatinine and BUN were assessed in the 24 h urine (Cobas Integra 400 plus, Roche^®^, Roche Diagnostics Inc.), and the urine volumes were measured in order to calculate creatinine and BUN clearance and the glomerular filtration rate, according to previously described protocols [48].

### 4.4. Serum, Kidney and 24 h Urine Lipid Peroxidation

Lipid peroxidation was determined by assaying the malondialdheyde (MDA) production by means of the thiobarbituric acid (TBA) test, as previously described [49]. Briefly: 100 μL of renal tissue supernatant, serum or urine (previously centrifuged to remove particulates) were incubated 1 h in a TBA solution. Samples incubated at 90 °C for 60 min. In this test, one molecule of MDA reacts with two molecules of TBA with the production of a pink pigment producing maximal absorbance at 532 nm. The concentration of MDA was calculated in respect to a calibration curve using 1,1,3,3-tetramethoxy propane as the external standard (range: 0.1–83.5 μM) and results were expressed as μM/g tissue for kidney and μM for the serum and urine evaluations.

### 4.5. Real Time Quantitative Polymerase Chain Reaction (RT-qPCR) in the Kidney

#### 4.5.1. Total RNA Isolation

The kidney, was stored in RNA later™ solution (Ambion, Austin, TX, USA). For RNA extraction, 10 mg of tissue were weighted and 450 μL of RLT Lysis Buffer was added, tissue disruption and homogenization for 2 min at 30 Hz was performed using a TissueLyser (Qiagen, Hilden, Germany). Tissue lysates were processed according to the RNeasy^®^ Mini Kit protocol (Qiagen, Hilden, Germany). Total RNA was eluted in 50 μL of RNase-free water (without optional treatment with DNAse). In order to quantify the amount of total RNA extracted and to verify RNA integrity (RIN, RNA Integrity Number), samples were analyzed using a 6000 Nano Chip^®^ kit, in the Agilent 2100 bioanalyzer (Agilent Technologies, Walbronn, Germany) and the 2100 expert software, following manufacturer’s instructions. The isolation yield was from 0.5 to 3 μg; RIN values were 6.0–9.0 and purity (A260/A280) was 1.8–2.0.

#### 4.5.2. Reverse Transcription

RNA was reverse transcribed with SuperScript™ III First-Strand Synthesis System for Real Time Polymerase chain Reaction (RT-PCR) (Invitrogen, Carlsbad, CA, USA). One microgram of total RNA was mixed with a 2× First-Strand Reaction Mix and a SuperScript™ III Enzyme Mix (Oligo(dT) plus Random hexamers). Reactions were carried out in a thermocycler Gene Amp PCR System 9600 (Perkin Elmer, Norwalk, CT, USA), 10 min at 25 °C, 30 min at 50 °C and 5 min at 85 °C. Reaction products were then digested with 1 μL (2 U) RNase H for 20 min at 37 °C and, finally, cDNA was eluted to a final volume of 50 μL and stored at −20 °C.

#### 4.5.3. Relative Gene Expression Quantification

Gene expression was performed using a 7900 HT Sequence Detection System (Applied Biosystems, Foster City, CA, USA). A normalization step preceded the gene expression quantification, using geNorm Housekeeping Gene Selection kit for Rattus norvegicus (Primer Design, Southampton, UK) and geNorm software (Ghent University Hospital, Center for Medical Genetics, Ghent, Belgium) to select optimal housekeeping genes for this study [50]. Real-time PCR reactions used specific QuantiTect Primer Assays (Qiagen, Hilden, Germany) with optimized primers for transforming growth factor beta-1 (QT00187796), proliferating cell nuclear antigen (QT00178647), mechanistic target of rapamycin (QT00180586), monoclonal antibody Ki-67 (QT00450786) and tumor protein p53 (QT00193522) as proliferative markers; vascular endothelial growth factor b (QT01290163) as angiogenic marker; interleukin 1 beta (QT00181657), nuclear factor kappa B (QT01577975), tumor necrosis factor (QT00178717), cyclooxygenase 1 (QT00187859), cyclooxygenase 2 (QT00192934) and *C*-reactive protein (QT00391650) as inflammatory markers. Endogenous controls were also used: glyceraldehyde-3-phosphate dehydrogenase (QT00199633), β-actin (QT00193473), topoisomerase I (QT01820861) together with a QuantiTect SYBR Green PCR Kit (Qiagen, Hilden, Germany) used according to manufacturer’s instructions. RT-qPCR reactions were carried out with: 100 ng cDNA sample, primers (50–200 nM) and 1× QuantiTect SYBR Green PCR Master Mix. Non template control reactions were performed for each gene, in order to assure non unspecific amplification. Reactions were performed with the following thermal profile: 10 min at 95 °C plus 40 cycles of 15 s at 95 °C and 1 min at 60 °C. Real-time PCR results were analyzed with SDS 2.1 software (Applied Biosystems, Foster City, CA, USA) and quantification used the 2^−ΔΔ^*^C^*^t^ method [51]. The results were obtained in CNRQ (calibrated normalized relative quantities) and then converted in percentage with the control group as reference.

### 4.6. Renal Pathology

H&E staining: Samples were fixed in Bock’s fixative and embedded in paraffin wax, and 4 μm thick sections were stained for routine histopathological diagnosis with H&E.

#### 4.6.1. Periodic Acid of Schiff Staining

PAS was used to evaluate and quantify the renal lesions. Samples were fixed in 10% neutral formalin, embedded in paraffin wax, and 4 μm thick sections were immersed in water and subsequently treated with a 1% aqueous solution of periodic acid, then washed to remove any traces of the periodic acid, and finally treated with Schiff’s reagent. All samples were examined by light microscopy using a Zeiss Microscope Mod. Axioplan 2 (Carl Zeiss Microscopy GmbH, München, Germany). The degree of injury visible by light microscopy was scored in a double-blinded fashion by two independent pathologists. Lesions were evaluated on the total tissue on the slide.

#### 4.6.2. Masson’s Trichrome Staining

Deparaffinize and rehydrate through 100% alcohol, 95% alcohol, 70% alcohol. Wash in distilled water. After that, re-fix in Bouin’s solution for 1 h at 56 °C to improve staining quality and rinse in running tap water for 5–10 min to remove the yellow color. Stain in Weigert’s iron hematoxylin working solution for 10 min and rinse in running warm tap water for 10 min. Wash in distilled water. Stain in Biebrich scarlet-acid fuchsin solution for 10–15 min and wash in distilled water. Differentiate in phosphomolybdic–phosphotungstic acid solution for 10–15 min and transfer sections directly to aniline blue solution and stain for 5–10 min. Rinse briefly in distilled water and differentiate in 1% acetic acid solution for 2–5 min and wash in distilled water. Finally, dehydrate very quickly through 95% ethyl alcohol, absolute ethyl alcohol, clear in xylene and mount with resinous mounting medium.

#### 4.6.3. Analysis of Lesions

Glomerular damage was assessed by evaluating mesangial expansion, the glomerular basement membrane, the capsule of Bowman thickening, nodular sclerosis, glomerulosclerosis, atrophy, and hyalinosis of the vascular pole. The analyzed tubulointerstitial lesions comprised inflammation, presence of hyaline cylinders, tubular basement membrane irregularity, tubular calcification, and the association of interstitial fibrosis and tubular atrophy (IFTA). The evaluation of vascular lesions was concentrated on arteriolar hyalinosis, arteriolosclerosis and arteriosclerosis. A semi-quantitative rating for each slide ranging from normal (or minimal) to severe (extensive damage) was assigned to each component. Severity was graded as absent/normal (0), mild (1), moderate (2), and severe (3). Scoring was defined according to the extension of the lesion (number of capsules): normal: 0%; mild: <25%; moderate: 25%–50%; severe: >50%. The final score of each sample was obtained by the average score observed in the individual glomeruli, in the considered microscopic fields. Tubular calcification was evaluated and graded by the same semiquantitative method. Regarding vascular lesions, arteriosclerosis was scored as 0 if no intimal thickening was present, as 1 if intimal thickening was less than the thickness of the media, and as 2 if intimal thickening was more than the thickness of the media and considering the worst artery on the slide. Using PAS, the rating was set for intensity and extension of staining, ranging from 0 (no staining) to 3 (intense and extensive staining), respecting tissue specificity scoring when adequate.

### 4.7. Kidney mTOR Protein Expression by Immunohistochemistry

Sections of paraformaldehyde fixed kidney tissues were processed by indirect immune detection technique with mousse and rabbit specific HRP/DAB detection IHC kit (Abcam, Cambridge, UK) using the primary antibody mammalian target of rapamycin (dilution 1:250; Millipore Co., Billerica, MA, USA). Diaminobenzidine (DAB) was used as chromogen. The protocol was executed according to the manufacturer’s instructions. An appropriate positive control was used in each staining run, and each slide was stained with a negative control obtained by omitting the primary antibody. Standard procedures were used for visualization and the staining was quantified using a semiquantitative scale (0–3) that evaluated both the intensity and area of staining. Intensity was graded as very low (0), low (1), moderate/mild (2) and high (3); staining area was graded as <25% (0), 25%–50% (1), 50%–75% (2) and >75% (3). All slides were reviewed independently by 2 investigators blinded to the data.

### 4.8. Statistical Analysis

Statistical analyses were performed using the GraphPad Prism^®^ for Windows (version 5.00, GraphPad Software Inc., La Jolla, CA, USA). The results are presented as means ± SEM. Comparisons between groups were performed using the Student’s *t*-test. Significance was accepted at *p* < 0.05.

## 5. Conclusions

CsA-induced nephrotoxicity is significantly aggravated over time and distinct mechanisms seem to underlie either short- or long-term nephropathy. Biochemical impairment (serum and urine creatinine and BUN contents and clearance, as well as kidney lipid peroxidation) starts after 3 weeks of treatment, but is aggravated with a longer exposure, while renal tissue lesions only appear after a chronic exposure, accompanied by remarkable kidney *mTOR* and *Mki67* over-expression, as well as MDA clearance increase, suggesting the presence of distinct pathways and biomarkers throughout the transition from CsA-induced renal dysfunction to nephrotoxicity. These findings reinforce two key aspects in post-transplant therapeutics: the need to identify better biomarkers of transition from CsA-induced dysfunction to nephrotoxicity to improve clinical decisions and the molecular justification for early substitution of CsA by other less nephrotoxic immunosuppressive agents, being mTOR inhibitors the most suitable choice, in order to reduce the risk of chronic allograft nephropathy, thus improving outcomes in transplanted patients.
